# Application of Graphene and its Derivatives in Detecting Hazardous Substances in Food: A Comprehensive Review

**DOI:** 10.3389/fchem.2022.894759

**Published:** 2022-07-01

**Authors:** Jinjin Pei, Ting Ren, Yigang Huang, Rui Chen, Wengang Jin, Shufeng Shang, Jinze Wang, Zhe Liu, Yinku Liang, A. M. Abd El-Aty

**Affiliations:** ^1^ Shaanxi Province Key Laboratory of Bio-resources, QinLing-Bashan Mountains Bioresources Comprehensive Development C. I. C., Qinba State Key Laboratory of Biological Resources and Ecological Environment, College of Bioscience and Bioengineering, Shaanxi University of Technology, Hanzhong, China; ^2^ Department of Pharmacology, Faculty of Veterinary Medicine, Cairo University, Giza, Egypt; ^3^ Department of Medical Pharmacology, Faculty of Medicine, Atatürk University, Erzurum, Turkey

**Keywords:** electrochemical detection, graphene derivatives, pigments, pesticides, toxins

## Abstract

Graphene and its derivatives have been a burning issue in the last 10 years. Although many reviews described its application in electrochemical detection, few were focused on food detection. Herein, we reviewed the recent progress in applying graphene and composite materials in food detection during the past 10 years. We pay attention to food coloring materials, pesticides, antibiotics, heavy metal ion residues, and other common hazards. The advantages of graphene composites in electrochemical detection are described in detail. The differences between electrochemical detection involving graphene and traditional inherent food detection are analyzed and compared in depth. The results proved that electrochemical food detection based on graphene composites is more beneficial. The current defects and deficiencies in graphene composite modified electrode development are discussed, and the application prospects and direction of graphene in future food detection are forecasted.

## Graphene and its Derivatives

**FIGURE 1:**
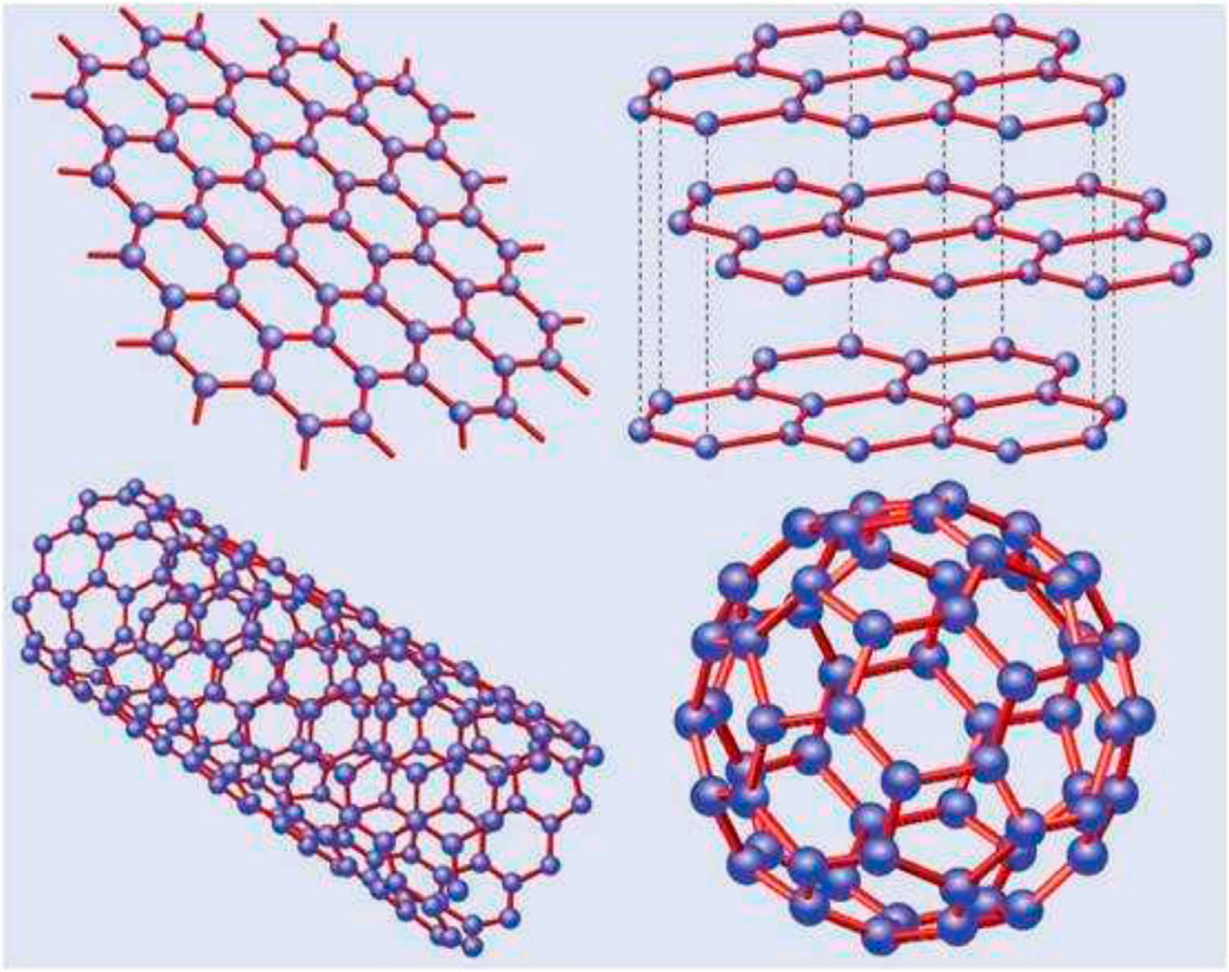
Basic structure of graphene (YARRIS et al., 2007).

Graphene is a single-atom 2D layer crystal atom with sp^2^ hybridization of carbon atoms, with a thickness of only 0.34 nm (Figure 1). The inner carbon atoms are arranged in a honeycomb lattice structure. According to different application requirements, the mechanical peeling method, SiC epitaxial growth method, Hummers’ method, and chemical vapor deposition (CVD) method are commonly used in carbon fabrication. The advantages and disadvantages of the methods mentioned previously vary. Graphene has remarkable physical and chemical properties such as high mechanical strength, excellent thermal and electrical conductivity, and a high specific surface area (theoretically, 2,630 m^2^/g for single-layer graphene) (Alwarappan et al., 2009; Balandin, 2011; Neto et al., 2009; Wang et al., 2009). It has significant application prospects in numerous fields, including the food industry, material manufacturing, energy, chemical industry, biological science, and medical medicine delivery.

**FIGURE 2:**
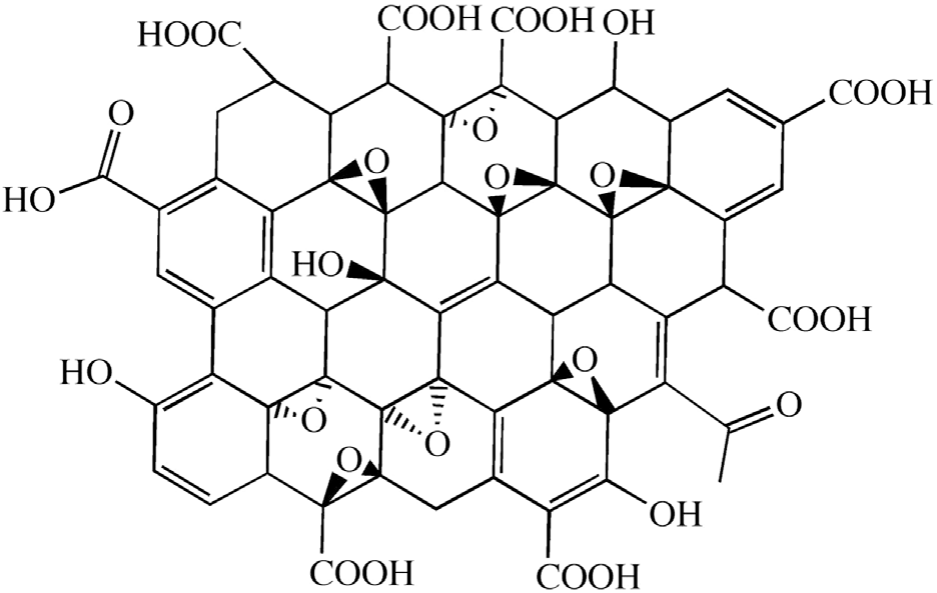
Structure of graphene oxide (Zhou et al., 2011).

Graphene oxide (GO), an oxygen-containing derivative of graphene, belongs to a branch of graphene research. Generally, graphene oxide is a material obtained by the multistep chemical or thermal reduction of graphene (Figure 2) (Dikin et al., 2007; Stankovich et al., 2007). Chemically synthesized GO is suitable for mass production, and the GO surface contains abundant oxygen-containing groups, which gives it greater thickness than graphene. Due to these functional groups, the GO surface is easily modified, with a fast electron transfer rate and good hydrophilic and biocompatibility properties (Dreyer et al., 2010; Liu et al., 2010; Zhou et al., 2009). The abundant oxygen-containing groups (such as -OH and -COOH) provide GO with strong hydrophilic ability. It can be dispersed in water or other organic solvents to form a stable suspension. It is also easy to modify GO through covalent or noncovalent interactions with organic small-molecule polymers (Wang et al., 2011). Recently, GO-based materials have gained increasing interest due to their excellent attributes.

Reduced graphene oxide (rGO) is an ideal support material for metal nanoparticles, which can be produced at a low cost and large scale. rGO sheets possess higher conductivity than GO sheets due to restoring the conjugated network in the rGO sheets. The oxygen-containing functional groups on rGO render it with an extremely high specific surface area, superior electronic conductivity, excellent mechanical strength, and elasticity (Sahoo et al., 2012; Kong et al., 2009). rGO is considered a promising material in the fabrication of electrochemical biosensors (Zhang L. et al., 2012; Kuila et al., 2011; Lu et al., 2012).

**FIGURE 3:**
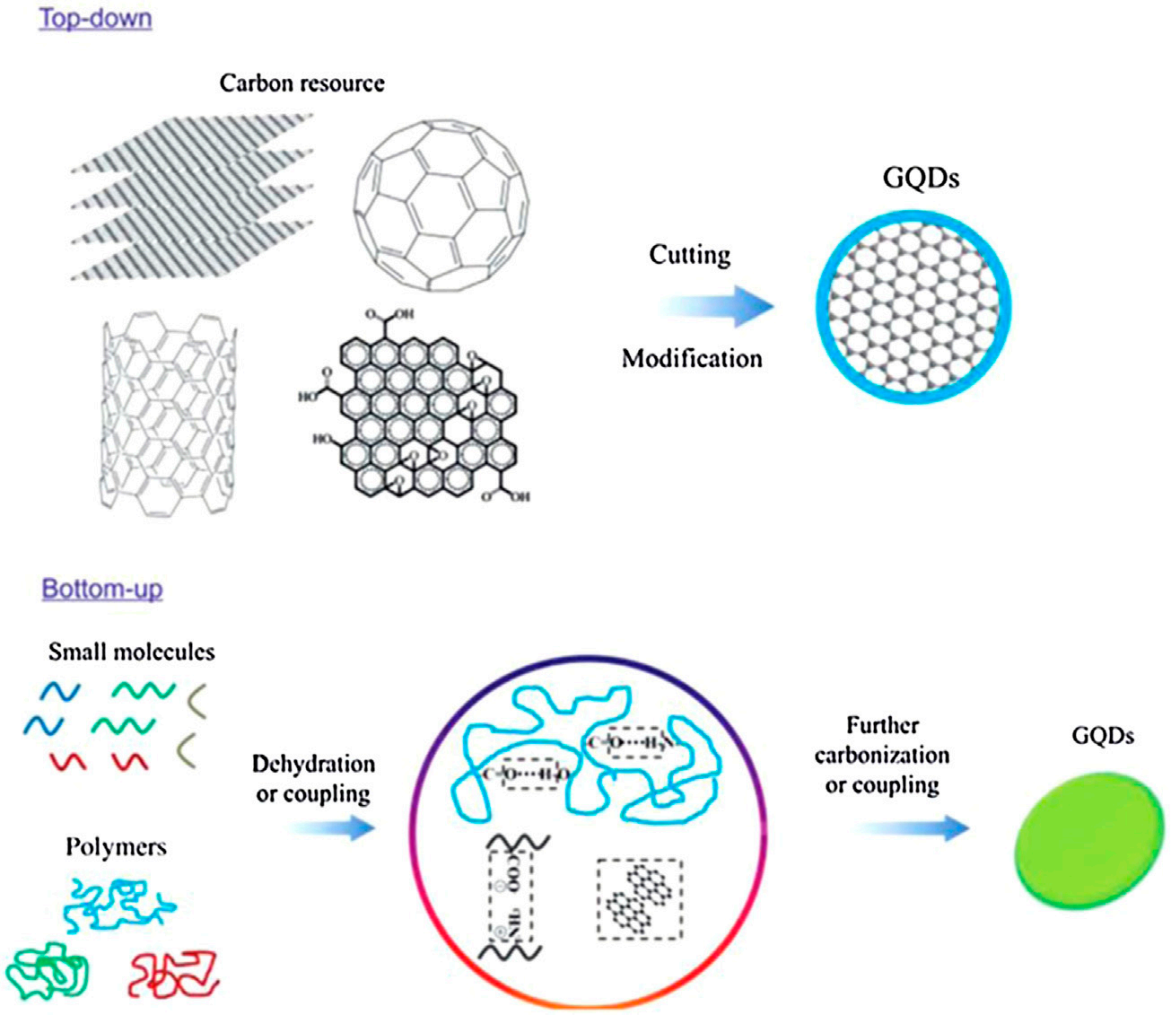
Two approaches to synthesizing GQDs: the “top-down” splitting from large molecules and “bottom–up” from small molecules (Zhu et al., 2017).

Graphene quantum dots (GQDs) are attractive nanomaterials consisting of a monolayer, or a few layers, of graphene with excellent and unique properties. Unlike graphene sheets, GQDs are 0D graphene segments that exhibit bandgap responsible for their unique optical and electrical properties. Due to their small size, GQDs display a quantum effect when produced using carbon-rich precursors such as fullerene, glucose, graphite, graphene oxide (GO), CNTs, and carbon fibers (CFs). Two main GQD synthesis methods are followed, namely, top–down and bottom–up methods, as shown in Figure 3 (Zhu et al., 2017). Such techniques are too complicated for the synthesis of conventional semiconductor quantum dots.

## Preparation of Graphene and Its Derivative

### Mechanical Peeling Method

Graphene sheets are removed directly from the surface of large graphite crystals by mechanical force and transferred to carriers such as silica. Monolayer graphene sheets can be obtained on substrates such as silica. This is the first known method to synthesize graphene using a top–down technique, by which layered graphene is split into several layered graphene sheets through repeated peeling. The obtained flakes varied greatly in size and thickness, and the product size could not be controlled. Therefore, it could not meet the industrial requirements of graphene. Entani et al. developed and modified these methods by synthesizing graphene on a SiO_2_ substrate. Their research showed that the resulting graphene structure was more homogenous than when using other methods.

### Epitaxy Growth Method

Under high vapor pressure, high temperature (usually >1400°C), and ultra-high vacuum pressure (usually <10^6^ Pa), silica atoms are volatilized, and the residual carbon atoms are arranged on the SiC surface to form graphene layers throughout the structure.

This method can result in large amounts of high-quality single graphene, but the SiC crystal is expensive, and the transfer of the produced graphene is challenging (Dreyer et al., 2010). Therefore, this method is mainly used to prepare graphene devices with SiC as the substrate.

### Hummers’ Method

The key idea of this method is forcibly damaging π and introducing oxygen-containing functional groups made of O and C atoms, such as COOH, -OH, and C–O–C. Subsequently, graphene experiences chemical, thermal, or electrochemical reduction to remove the oxygen-containing functional groups to obtain reduced graphene oxide (reduced graphene oxide, rGO) (Zhou et al., 2009; Zhou et al., 2011). Currently, the most used chemical oxidation method is Hummers’ method. In this method, inorganic strong protic acids (such as concentrated sulfuric acid, concentrated HNO_3_, or a mixture) are used to treat the raw graphite. Small molecules of a strong acid are inserted in between the graphite layers, followed by strong oxidants such as KMnO_4_ and KCIO_4_ (Lee et al., 2016). Finally, hydrogen peroxide is used to remove any excess KMnO_4_. After filtration, diluted hydrochloric acid and ultra-pure water are used to clean the products and obtain GO (Dreyer et al., 2010). The degree of graphite oxidation depends on the selected method and reaction conditions. This method is currently the most commonly used in laboratory GO preparation, but it often fails to obtain high-quality graphene (Liu et al., 2010).

### Chemical Vapor Deposition

The graphene prepared *via* CVD is of high quality and is expected to meet the application requirements of transparent conductive films. It is widely used in the large-scale industrial preparation of semiconductor materials for thin-film transistors.

## Application of Graphene in Food Detection

As a branch of chemistry, electrochemical analysis has been verified by many practical and theoretical experiments. It is an analytical method to determine the composition and content of an object by directly measuring different electrical signal parameters, such as the current potential conductance, according to the electrochemical characteristics of different substances. Electrochemical detection methods have the advantages of low cost, small working space, fast speed, high sensitivity, and high accuracy when compared with other food detection methods. Therefore, the research, application, and promotion of electrochemical detection in food quality and related areas can play an essential role in promoting future food quality detection and standards. As an eminent factor in materials science in the 21st century, graphene has been widely used according to its excellent properties (Novoselov et al., 2012). Applying single graphene or graphene-based composite materials in the chemical modification of electrodes also shows significant development and research value. In addition, graphene and some of its derivatives, including graphene oxide (GO) and reduced graphene oxide (rGO), have been applied to detect substances in different food samples. These include colorants in food and drinks (He et al., 2018a; Qiu et al., 2016), heavy metal ions (Xia et al., 2015), antibiotic residues (Liu et al., 2017), dopamine, rutin from human samples (Yang and Li, 2014; He et al., 2018b; He et al., 2019a), pesticide residues (Liu et al., 2011), and 4-nitrophenol (He et al., 2019b) from environmental contaminants. GO and rGO showed great promise in developing chemically modified electrodes (Shao et al., 2010).

### Detection of Food Coloring

The synthetic pigments that commonly appear in food are azo pigments (tartrazine, Allura Red, amaranth, carmine, and sunset Yellow). Azo dyes account for more than half of the global synthetic dye production. Azo pigments exhibit excellent color performance, and desired colors can be obtained by adjusting the type and proportion of the azo components, creating a wide variety of colors. The excessive intake of azo dyes may be harmful to human health. For example, sunset yellow and tartrazine contain groups such as azo and aromatic rings in their structure, which can induce allergies, asthma, migraines, and cancers (Somasundaram, 2014; Ye et al., 2013). Existing analytical methods for the detection of azo pigments mainly involve high-performance liquid chromatography (HPLC–MS) (Qiu et al., 2016), capillary electrophoresis (Zhao et al., 2014), surface-enhanced Raman spectroscopy (SERS) sensors (Xie et al., 2012), fluorescence (Yuan et al., 2016), and thin-layer chromatography (Soponar et al., 2008). Some of these methods are complex and do not meet economic, efficient, and convenient detection demands. Presently, electrochemical analysis methods using modified electrodes have been used to detect food additives such as citron yellow and sunset yellow as they are convenient, fast, sensitive, and highly selective (Table 1) (Qiu et al., 2016; Gan et al., 2013a; Wang and Zhao, 2015a; Wang and Zhao, 2015b).

**TABLE 1:** Applications of graphene in detecting pigments and heavy metal ions.

Food Colorants	Material/electrode used	Method	Limit of detection	Linear range	Reference
Sunset yellow	MnO_2_ NRs-ERGO- GCE	CV and SDLSV	2.0 nM	0.01–2.0 µM, 2.0–10.0 µM and 10.0–100.0 µM	Ding et al., 2019
Sunset yellow; tartrazine	Carbon-ceramic electrode of graphene nanoplatelet	DPV	7.3 × 10^–8^ M; 8.1 × 10^–8^ M	1 × 10^−7^–1.5 × 10^–5^ M; 1 × 10^−7^–2×10^–5^ M	Majidi et al. (2015)
Sunset yellow	Cv	ILRGO-Au/GCE	5.2 × 10^−10^M	4.0 × 10^−9^–2×10^−6^ M	Wang et al. (2015)
Sunset yellow	DPV	rGO/CPE	27 nM	0.05–10 µM	Elham et al. (2020)
SY, Tz	GN/TiO_2_-CPE	Square wave voltammetry (swv)	6.0 nM, 8.0 nM	0.02–2.05 µM, 0.02–1.18 µM	Gan et al. (2013b)
Sudan I	(graphene/β-CD/PtNPscomposite modified electrode); graphene/β-CD/PtNP modified electrode	DPV	1.6 nM	0.005–68.68 μM	Palanisamy et al. (2017)
Sudan I	Ag-CuNP/rGO/GCE	CV	0.4 nM	1.0 nM-10 µM	Yao et al. (2016)
Sudan I	GMGCE	New voltammetric method	4.0 × 10^−8^mol L^−1^	7.50 × 10-8 mol L^−1^–7.50 × 10^−6^ mol L^−1^	Ma et al. (2013a)
Allura red	CV and DPV	PDDA-Gr-Ni/GCE	8.0 nmol/L	0.05–10.0 mmol/L	Yu et al. (2016)
Allura red	CV SWSV	IL-GO-MWCNT-GCE	5.0 × 10^−10^ mol/L; 3.0 × 10^−9^ mol/L	8.0 × 10^–10^–5.0 × 10^−7^ mol/L; 5.0 × 10^−9^–4.5 × 10^−7^ mol/L	Wang et al. (2015)
Allura red	CV and DPV	PDDA-Gr-Ni/GCE	8.0 nmol/L	0.05–10.0 mmol/L	Yu et al. (2016)
Amaranth	GNM/GCE	CV	7.0 × 10^–10^ M	5.0 × 10^–9^–1.0 × 10^–6^ M	Wang et al. (2018b)
Amaranth	DPV	Fe_3_O_4_/rGO	50 nM	0.05–50 µM	Han et al. (2014)
Heavy metal ions					
Pb(II), Cd(II), Zn(II), Cr(III), and Mn(II) and Fe(III)	DMSPE/ICP-OES	GO	Pb(II)-0.25 ng ml^−1^	—	Feist and Sitko, (2019)
			Cd(II)-0.06 ng ml^−1^		
			Zn(II)-0.16 ng ml^−1^		
			Cr(III)-0.06 ng ml^−1^		
			Mn(II)-0.12 ng ml^−1^		
			Fe(III)-0.21 ng ml^−1^		
Pb(II)	BGO-SLPE/FAAS	BGO	0.84 μg L^−1^	—	Wang et al. (2018a)
Hg(II)	ICP-OES	Fe_3_O_4_@GO/2-PTSC	0.0079 μg L^−1^	—	Keramat and Zare-Dorabeni (2017)
Pb2+; Cd2+	DPASV	GC–O–GO	Pb^2+^-0.25 pM; Cd^2+^-0.28 pM。	—	Yavuz et al. (2016)
Heavy metals	FAAS	Amine-functionalized graphene nanosheet	Cd(II)-0.03, Cu(II)-0.05, Ni(II)-0.2, Zn(II)-0.1, Pb(II)-1 μg L^−1^	—	Behbahani et al. (2014)
Cd(II)	FAAS	Dipyridyl-functionalized graphene nano-sheet	0.19 ng ml ^−1^	—	Karimi et al. (2014)
Mn(II) Fe(III)	FAAS	MPPC chelates on GO	Mn(II)-145 ng.L^−1^ Fe(III)-162 ng. L^−1^	—	Pourjavid et al. (2014)
Pb2+	SWASV	rGO-SPCE	1 ppb (S/N = 3)	—	Jian et al. (2013)
Trace heavy metals	SWASV	RGO-N/Si	Cd^2+^-1.69 nM, Pb^2+^-0.39 nM, Cu^2+^-2.16 nM	—	Lee et al. (2016)
Toxic heavy metals (Cd, Hg, and Pb)	DPASV	PG/GCE	Cu^2+^-0.024 µM, Cd^2+^-0.015 µM	—	Yi et al. (2019)
			Hg^2+^- 0.032 µM, -S/N ≥ 3)		
Pb(II)	SWV	PGA/rGO	0.06 μg/L	—	Feminus and Deepa, (2019)
Pb(II)	SWV	NG-PTCA-Thi-Au	0.42 pM (S/N = 3)	—	Ma et al. (2019)
Cd(II); Pb(II)	SWASV	GC/ErGO	Cd(II)-15 μg/L; Pb(II)-15 μg/L	—	Pizarro et al. (2019)
Cu(II)	FAAS	AF-Fe_3_O_4_– GO-based MSPE	0.9 μg L^−1^	—	Bahar and Karami, (2015)
Zn(II)	FAAS	IIP-GO/Chm	0.09 μg L^−1^	—	Kazemi et al. (2017)
Cd2+	ICP-OES.	(MGO@Azo-phenol	0.4 μg L^−1^	—	Sa’adi and Es haghiet al. (2019)

CV, cyclic voltammetry; DPV, different pulse voltammetry; LSV, linear sweep voltammetry; FAAS, flame atomic absorption spectrometry; ICP-OES, inductively coupled plasma optical emission spectrometer; SWV, Schutz–Werke–Verzeichnis; DPASV, differential pulse anodic stripping voltammetry; SWASV, square wave stripping voltammetry.

Gan et al. used a carbon paste electrode, modified by graphene and mesoporous TiO_2_, to develop a rapid and low-cost method without any sample pre-treatment required to detect sunset yellow and tartrazine yellow colorants, with the detection limits of 6.0 and 8.0 nM(Gan et al., 2013b), respectively. Ding et al. (2019) studied electrochemically reduced graphene oxide (ERGO) nanoflake-decorated MnO_2_ nanorods (MnO_2_ NRs) with a modified glassy carbon electrode (GCE) surface. When compared with standard GCE, a well-defined sunset yellow (SY) oxidation peak was observed at the MnO_2_ NR-ERGO/GCE. It had a detection limit of 2.0 nM and a good linear response to the SY in the ranges of 0.01–2.0 µM, 2.0–10.0 µM, and 10.0–100.0 µM. This method was applied to detect SY in soft drinks and obtained satisfactory results (Ding et al., 2019). Vatandost et al. used natural tea extracts to obtain modified rGO on a carbon paste electrode surface (rGO/CPE). Since rGO/CPE has a large surface area, it has a strong enhancement effect on the electrochemical oxidation of SY, with a wide linear response range of 0.05–10 M and a detection limit of 27 nM (Elham et al., 2020). Wang et al. used a graphene nanometer material (GNM)–modified electrode, which exhibited a significantly enhanced electrochemical amaranth signal. It showed a wider linear response range from 5.0 × 10^–9^ to 1.0 × 10^–6^ M. It also possessed a low detection limit of 7.0 × 10^–10^ M at a signal-to-noise ratio of 3 (Wang et al., 2018). Palanisamy et al. fabricated graphene/β-CD/PtNP-modified electrodes through platinum nanoparticles (PtNPs) decorated with graphene-β-cyclodextrin (graphene/β-CD)–modified electrodes. Studies on the electrode cyclic voltammetry determination of different modified electrodes on the electrochemical behavior of Sudan I indicated that the electric catalytic activity of graphene/β-CD/PtNP-modified electrodes on Sudan I was higher than that of other modified electrodes. With a detection linear response range of 0.005–68.68 µM and a detection limit of 1.6 nM, the sensor was used for monitoring Sudan I–adulterated food samples (chili powder, chili sauce, and tomato sauce) and achieved satisfactory results in practical tests (Palanisamy et al., 2017).

### Detection of Heavy Metals in Food

The excessive toxic heavy metal content is hazardous to human health, causing illnesses such as renal dysfunction, cancer, cardiovascular, and cerebrovascular diseases (Kadıoğlu et al., 2010). With industrial development, the frequent pollution accidents in the environment make food unsafe, and excessive toxic heavy metals have become one of the most severe threats to human health. Therefore, it is essential to develop a rapid, sensitive, and simple method for detecting heavy metal ions using analytical techniques, such as atomic fluorescence spectroscopy, inductively coupled plasma optical emission spectrometry (Ozbek and Akman, 2016), and induced plasma-atomic emission spectrometry (Feist and Mikula, 2014).

Graphene and its derivatives with fast electron transmission power, strong adsorption performance, and easy chemical modification are used for the electrochemical detection of heavy metals (Table 1) (Li et al., 2013). This operating instrument has attracted wide attention due to its low cost, small footprint, fast detection speed, high sensitivity, and high accuracy (Aragay and Merkoci, 2012; Zhu et al., 2014). GO possesses abundant active functional groups (such as carboxyl, hydroxyl, and epoxy), making it an excellent adsorbent for environmental applications. Despite the excellent properties of GO, its high hydrophilic nature, extensive agglomeration, and difficulty in separation from treated wastewater are considered drawbacks. In addition, the oxygen atom in the carboxyl group is categorized as a hard ligand group with less affinity for pollutants such as heavy metals (Chen et al., 2015; Zhao et al., 2016; Cui et al., 2015b). Consequently, the modification of GO through the introduction of other functional groups is the subject of ongoing research. The detection and adsorption of heavy metals were altered by modifying GO by introducing organic functional groups from multifunctional materials (Hu et al., 2015). This functionalization enhances the excellent properties of GO and increases its adsorption capacity for the removal of heavy metal ions (Gul et al., 2016).

Although zinc is one of the most important essential trace elements, a lack in the human body will lead to various diseases. However, excessive zinc intake is toxic and harmful to the body, causing various adverse reactions. Therefore, it is of great significance to determine the sensitivity of zinc in different food matrices (Feist and Mikula, 2014). Kazemi et al. proposed an innovative method for the selective extraction and determination of zinc. A novel zinc-imprinted polymer was synthesized by the co-precipitation of graphene oxide/magnetic chitosan nanocomposites. After optimizing the extraction process’s technical parameters, the adsorbent’s maximum adsorption capacity was 71.4 mg g^−1^, and the detection limit was 0.09 μg L^−1^ (Kazemi et al., 2017).

During the past decade, many researchers have combined other materials to form new graphene-based nanomaterials that can be used to detect heavy metal concentrations (Table 1). Palisoc et al. prepared graphene/gold nanoparticles (AuNPs)/hexamine mineral acid [Ru(NH_3_)_6_] 3^+^/Nafion *via* the drop coating method on a glassy carbon electrode (GCE), which successfully determined trace amounts of Pb(II), Cu(I), Cu(II), Sn(IV), and Hg(II) in canned food samples. Pb^2+^, Cd^2+^, and Cu^2+^ exhibited the highest sensitivity by anodic stripping voltammetry (ASV). There was an excellent linear relationship between heavy metal concentration and peak current, and the detection limit of Pb^2+^ was 0.74 ppb, that of Cd^2+^ was 37 ppb, and that of Cu^2+^ was 945 ppb (Palisoc et al., 2019). Wang et al. improved the extraction efficiency of Pb(II) in food samples through the synergistic effect of the BGO membrane (solid extraction) and organic solvent (liquid extraction). After optimizing the BGO membrane composition parameters, pH, elution agent types, elution time, sample volume, and other experimental conditions showed no obvious influences on different competitive ions. Under the optimal experimental conditions, the minimum detection limit was 0.84 μg L^−1^ and the precision was 4.65%. The method has been successfully verified based on the analysis of detecting Pb(II) added to food samples. Karthik used electron microscopy (HR-SEM), transmission electron microscopy (TEM), X-ray diffraction (XRD), and UV–Vis-NIR Fourier-transform infrared spectroscopy (FT-IR) to analyze cobalt-doped zinc oxide/reduced GO (Co: ZnO/RGO) nanorods, obtained by chemical co-precipitation (Wang J. et al., 2018). It was found that the electrode prepared with Co: ZnO/RGO nanorods had good sensitivity to Cd(II) and Pb(II) ions with excellent electrocatalytic oxidation performance, and the detection limit was 0.94 g/L (Cd(II)) and 0.83 g/L (Pb(II)), with concentrations ranging from 10 to 90 g/L (Karthik and Thambidurai, 2017).

### Detection of Food Pesticides

Organophosphate pesticides (OPs) have been widely used in agricultural production due to their wide control range, low cost, and high insecticidal efficiency. Organophosphates are neurotoxins that inhibit acetylcholinesterase activity, causing acetylcholine accumulation and neurotoxicity (Gu et al., 2012). In the European Union, it is already banned from being used on crops and is only allowed under strict limitations. Taking the organophosphate insecticide phoxim as an example (Chao and Chen, 2014), it is mainly used to prevent underground pests, particularly the Lepidoptera pests affecting peanuts, vegetables, and other crops. Various methods have been developed to identify phoxim, such as high-performance liquid chromatography (HPLC) (Hamscher et al., 2007; Liang et al., 2005; Liang et al., 2006; Liu et al., 2009; Lv et al., 2009), liquid chromatography (Guo et al., 2005; Lee et al., 2010), gas chromatography (Qu et al., 1997), near-infrared spectroscopy (Gu et al., 2012; Shen et al., 2009), and spectrophotometry (Ni et al., 2007). These methods require expensive equipment, large amounts of organic solvents, or are time-consuming. Electrochemical sensors have attracted increasing attention in recent years due to their convenience, speed, high sensitivity, and selectivity (Table 2) (Huang et al., 2011; Jin et al., 2011; Keawkim et al., 2013; Wang et al., 2014). Using graphene-modified glassy carbon electrodes, Chao and Chen established an electrochemical method to directly determine phoxim traces in vegetables, meat, and egg samples. The Gr/GCE combined with linear scanning voltammetry (LSV) was successfully applied to determine phoxim in food samples such as cauliflower, lamb, and quail eggs. This method has a very sensitive nanomolar detection limit for phoxim, providing an important detection tool (Chao and Chen, 2014). Mani et al. reported an effective electrochemical sensor using NbC@Mo nanocomposite for various pesticides (i.e., fenitrothion). This study proved that NbC@Mo holds a higher electrochemical active area and improved electrocatalytic property toward FTN. A DPV-sensing platform displayed eminent electroanalytical parameters, such as a wide linear range (0.01–1889 µM) and low detection limit (0.15 nM) (Govindasamy et al., 2018). Furthermore, Mani et al. described a reproducible and reliable screen-printed carbon electrode (SPCE) modified with graphene oxide nanoribbons (GONRs) for sensitive determination of methyl parathion. The sensor exhibited two linear ranges: (1) 100 nM–100 μM, with a sensitivity of 1.804 μAμM^−1^ cm^2^, and (2) 100–2,500 μM, with a sensitivity of 0.8587 μAμM^−1^ cm^2^. The detection limit was 0.5 nM (S/N = 3). The method successfully determined methyl parathion in ugli and tomato fruits, beetroot, and broccoli, indicating its practical applicability (Govindasamy et al., 2017a).

**TABLE 2:** Applications of graphene in detecting pesticides.

Pesticide	Type	Linearity range	LOD	Example	Ref
Phoxim	Gr/GCE sensor	5.97–5,966 μg L^−1^	2.39 μg L^−1^	Vegetable, meat, and eggs	Lv et al. (2009)
Phoxim	LC–MS	0.02–1.0 mg kg^−1^	Not available	Eggs	Lv et al. (2009)
Phoxim	Near-infrared spectrometry	1–100 mg L^−1^	1 mg L^−1^	Water	Gu et al. (2012)
Phoxim	Chi/AChE/SnSe_2_/GCE biosensor	8–5,120 μg L^−1^	4 μg L^−1^	No real sample analyzed	Zhang et al., 2012a
Carbofuran	Hemin-complex/graphene	5.6 × 10^−6^–9.5 × 10^−5 ^mol/L	9 × 10^−9 ^mol/L	Carrots	Wong et al. (2014)
Carbofuran	ECV (AChE/Fe_3_O_4_)	5.0 × 10^−9^–9.0 × 10^−8 ^mol/L	3.6 × 10^−9 ^mol/L	—	Jeyapragasam and Saraswathi. (2014)
Carbofuran	CNPPE	0.5 × 10^−7^–4.4 × 10^−7 ^mol/L	0.5 × 10^−7^	—	Samphao et al. (2013)
Carbofuran	(TPN/Fe_3_O_4_	0.5–500 μg L^−1^	0.17 μg L^−1^	Cucumber, tomato, and tap water	Shahrebabak et al. (2019)
	NPs/GO				
Neonicotinoid pesticides	HPLC–DAD	0.5–100 μg L^−1^	0.08–0.1 μg L^−1^	Pear and tomato	Ma et al. (2013b)

In the early 1950s, methylcarbamate was introduced as a pesticide and is still used today (Wong et al., 2014). This is one of the most toxic carbamate insecticides used to control insects on various field crops, including potatoes, corn, carrots, and soybeans. Methylcarbamate is a systemic pesticide absorbed through plant roots and distributed to their organs, where the pesticides accumulate over time (Otieno et al., 2010). Classical analytical techniques such as gas chromatography (GC), high-pressure liquid chromatography (HPLC), and mass spectrometry (MS) are known to be sensitive and standardized techniques for the detection of carbofuran (Filho et al., 2010; Vera-Avila et al., 2012; Atrache et al., 2013). Electrochemical sensors, specifically chemically modified electrodes, have become of interest to researchers due to their portability, simplicity, minimal cost, and short analysis time (Table 2) (Wong et al., 2013; Zen et al., 2003). Wong et al. (2014) developed a technique for the sensitive and selective analysis of carbofuran pesticides based on a carbon paste electrode modified with hemin complex and graphene oxide. The selective detection of carbofuran was confirmed, and when the sensor was applied to food samples, the recovery results were close to 100%, similar to the HPLC method (Wong et al., 2014).

Methyl paraoxon (MOX) is a highly toxic organophosphate pesticide. It has been recently reported that MOX can enter the human body through ingestion, inhalation, or dermal penetration. Due to its high nondegradability, it can bind to fruits and vegetables. When consumed, it can impose sub-chronic and chronic diseases by inhibiting acetylcholinesterase in human metabolism. Umamaheswari et al. reported the detection of non-enzymatic electrochemical sensors based on the 3D porous phase graphene oxide sheet–encapsulated chalcopyrite (GOS@CuFeS_2_) nanocomposite. As-synthesized GOS@CuFeS_2_ nanocomposite film screen-printed carbon-modified electrode (SPCE) displays excellent electrocatalytic ability toward MOX. Under optimized working conditions, the modified electrode provides a linear response range from 0.073 to 801.5 µM. The detection limit was obtained as 4.5 nM. The sensor displayed outstanding sensitivity at 17.97 μA μM^−1^ cm^−2^ 35. The GOS@CuFeS_2_ nanocomposite–modified electrode shows greater real-time practicality in actual vegetable samples (Rajaji et al., 2019a).

### Detection of Food Toxins

One of the most harmful food contaminants is aflatoxin (AFT) (Conradt et al., 2015). Aflatoxin mainly exists in corn, soybean, peanut, grain, wheat, and other agricultural products and seriously affects food safety. AFB1 is classified as a group I carcinogenic compound according to the International Agency for Research on Cancer (IARC) (Liu et al., 2006). Bhardwaj et al. deposited GQDs *via* chemical synthesis on indium tin oxide (ITO)–coated glass substrates through electrophoretic deposition. The AFB1 monoclonal antibody was covalently fixed on the deposition electrode GQDs/ITO (Table 3). The detection limits of the standard and contaminated samples were 0.03 ng ml^−1^ and 0.05 ng g^−1^, respectively, which falls below the maximum acceptable limit stipulated by the European Union. This suggests that this method possesses a potential application value in detecting AFB1 in food (Bhardwaj et al., 2018). Srivastava et al. studied the fabrication of a highly sensitive label-free biosensor based on a graphene oxide platform to detect aflatoxin B1 (AFB1). Electrochemical impedance spectroscopy (EIS) detected the AFB1 concentration range. The impedimetric sensing response of immunoelectrodes as a function of AFB1 concentration demonstrated a wide linear detection range (0.5–5 ng/ml), high sensitivity (639 Ω ng^−1^ ml), improved detection limit (0.23 ng ml^−1^), and good stability (5 weeks) in label-free detection (Srivastava et al., 2014). Pumera described the application of graphene in biosensors used as biomolecular labels, including a bio-field effect transistor (Pumera, 2011). Reduced GO (rGO) is a promising electrochemical biosensor material due to its biocompatibility and the presence of oxygen-containing functional groups (especially carboxyl groups) (Pumera, 2010; Zhang C. et al., 2012; Kuila et al., 2011; Lu et al., 2012). Srivastava et al. (2013) synthesized chemically active rGO and deposited it onto an indium tin oxide (ITO)–coated glass substrate *via* electrophoretic deposition. The sensing results of the anti-AFB1/RGO/ITO––based immunoelectrode obtained as a function of aflatoxin concentration showed high sensitivity (68 mA ng^−1^ ml cm^2)^ and an improved detection limit (0.12 ng ml^−1^) (Srivastava S Kumar et al., 2013).

**TABLE 3:** Applications of graphene in detecting toxins.

Toxin	Material type	Method	Limit of detection (LOD)	Example	Ref
AFB1	BSA/anti-AFB1/GQDs/ITO	EIS	0.05 ng g^−1^	Corn	Bhardwaj et al. (2018)
Acrylamide	Fe_3_O_4_@G-TEOS-MTMOS RP-MSPE	GC–MS	0.061–2.89 mg kg^−1^	Boiled potato and fried potato with bright-fleshed, sweet potato, snack, banana chips, eggplant, and potato chips	Nodeh et al. (2018)
	RP-MSPE clean-up using				
	Fe_3_O_4_@G-TEOS-MTMOS				
Nitrites	Pd/Fe_3_O_4_/polyDOPA/RGO	CV	0.5 µM	Yellow	Zhao et al. (2017)
				River water and sausage extract	
Kanamycin	RGO-based fluorescent aptasensor	Fluorescence	1.0 × 10^−12^ M	Blood serum and milk	Ha et al. (2017)
Hydrazine and nitrite	CoHCF-rGO/GCE	DPV	0.27 µM-nitrite; 0.069 µM-hydrazine	Pickled food; water, and well water	Luo et al. (2015)
AFB1	GO/Au	EIS	0.23 ng ml^−1^	—	Srivastava et al. (2014)
AFB1	BSA-anti-AFB1/RGO/ITO	CV	0.15 ng ml^−1^	—	Srivastava S Kumar et al. (2013)
Maltol	SnO_2_@C@GO/GCE	SWV	12 nM	Biscuits, beer, wine, and juice	Gan et al. (2017)
Maltol	PMB/Gr/GCE	CV	6.50 × 10^–8^ mol L^−1^	Cake, beer, and cola	Ma et al. (2014)
AFB1	CdTe QODS (fluorescence-based)		6.25 × 10^−3^ng ml^−1^	—	Zekavati et al. (2013)
OTC, TC, DC, and CTC	E-spun-GO/PANCMA-NFs	HPLC–FLD	20.4–44.8 μg/kg	Chicken muscle, liver, and kidney	Weng et al. (2019)
NSAIDs	Fe_3_O_4_-G	LC–MS/MS)	0.1–50 μg L^−1^	Swine, chicken, and bovine	Wang et al. (2019)
Chloramphenicol (CAP)	Eu_2_O_3_/GO	Amperometry	1.32 nM	Milk and honey	Rajaji et al. (2019b)
Sulfadiazine	MIP/NiCo_2_O_4_/3D graphene	DPV	0.169 ng/ml	Milk	Wei et al. (2019)
Sulfadimidine					
FQs	Magnetic graphene (MG)	HPLC-UV	0.05–0.3 ng/g	Bovine milk, chicken muscle, and egg	He et al. (2017)
FQs	Graphene oxide	HPLC–FLD	0.0045–0.0079 ng/g	Chicken samples	Fan et al. (2015)

Maltol, chemically known as 3-hydroxy-2-methyl-4H-pyran-4-one, is a natural flavor enhancer widely used in food (cake, beer, and drinks). Maltol is banned in children’s food by both the FDA and China’s national food safety standards because of its potential to harm health and is also banned in Europe (Ma et al., 2014). Therefore, the detection of maltol in food is crucial. Ma et al. (2014) set up a rapid method of detecting maltol, where the electrochemical behavior of maltol at the modified electrode was studied by cyclic voltammetry (Table 3). The method performed well in terms of linearity (r = 0.9981 and 0.9955), recovery (97.99.3%), reproducibility (relative standard deviation 3.1%, *n* = 6), and stability. Subsequently, it has been successfully applied in maltol analysis in various foods (Ma et al., 2014). Gan’s study proposed an ideal cheap voltammetric method to determine maltol in complex food matrices. SnO2@C@Go nanocomposite is a novel electrochemical reinforced material used to prepare maltol in food electrochemical detection platforms, with an excellent linear range (0.08–10 µM) and low detection limit (12 nM). This detection method extends the application range of semiconductor materials and sheds some light on the fusion of electrochemical technology and analytical methods (Gan et al., 2017).

For highly carcinogenic nitrites (Absalan et al., 2015), it is essential to develop a simple method to detect and monitor their levels in drinking water, cured food, and environmental systems (Wang et al., 2017; Schierenbeck and Smith, 2017). Zhao et al. (2017) synthesized a Pd/Fe_3_O_4_/polyDOPA/RGO composite material using a green method, and its electrocatalytic activity on nitrite oxidation was excellent (Table 3). A modified glassy carbon electrode measured the nitrite, and the amperometric response results demonstrated a wide linear range of 2.5–6470 µM and a low detection limit of 0.5 µM. Moreover, the sensor could effectively monitor the change in the nitrite content in the rapid decay of cabbage. In addition, the monitoring results showed that the nitrite content reached its peak within 1 day of corrosion and decreased to a lower level after 3 days, which was consistent with the ion chromatography trend (Zhao et al., 2017).

Acrylamide (2-propenamide) is found in carbohydrate-rich food cooked under high temperatures. Nodeh et al. (2018) successfully cleaned and measured acrylamide in various foods using a Fe_3_O_4_@G-TEOS-MTMOS RP-MSPE method and compared it with previous TEOS-MTMOS d-SPE and other published works. The Fe_3_O_4_@G-TEOS-MTMOS RPMSPE method showed low LODs (0.061–2.89 μg kg^−1^) and high relative recovery (82.70–105.97%) (Table 3) (Nodeh et al., 2018).

Rajaji et al. (2020) synthesized a super-active electrocatalyst of Bi_2_Te_3_@g-C_3_N_4_ BNs to quantify food toxic chemicals in meat samples. As modified, the Bi_2_Te_3_/g-C_3_N_4_ BN-modified electrode exhibits excellent electrochemical activity toward food toxic ractopamine (RAC) with high-sensitive (L.R: 0.015–456.4) and nanomolar detection limit (LOD: 1.77 nM) (Rajaji et al., 2020).

Govindasamy et al. (2019) developed a sensitive electrochemical (voltammetric; DPV) sensor for the determination of coccidiostat drug (roxarsone) based on the use of an SPCE (screen-printed carbon electrode) modified with tungsten disulfide nanosheets (WS_2_ NSs). Features including (a) a wider linear range (0.05–490 μM), (b) a nanomolar detection limit (0.03 μM), and (c) high sensitivity (29 μA μM^−1^ cm^−2^) have been recorded. It also yields high accuracy and good recovery (Govindasamy et al., 2019).

### Detection of Food Antibiotics

Abuse of antibiotics will lead to excessive amounts in livestock, poultry, meat, and eggs. Excessive drug and antibiotic residue in livestock and poultry products threatens public health and restricts the breeding industry’s sustainable development (Hao et al., 2014; Liu et al., 2017). There are significant differences between countries and species regarding the maximum residue limits of some veterinary drugs. Developing a reliable, highly sensitive detection method for antibiotics is conducive to better food quality supervision and guaranteed human food safety and benefits the country’s export trade in agricultural products. The SP–HPLC–FLD method of E-spun GO/pancma-NF proposed by Weng et al. has been successfully applied to TC analysis in chicken tissue samples (Table 3). The adsorption of tetracycline antibiotics (TCs) in chicken samples was studied by electrospun graphene oxide–doped poly (acrylonitrile-co-maleic acid) nanofibers (E-spun-GO-PANCMA-NFs), as a new adsorbent for solid-phase extraction (SPE). The results showed good linearity in the range of 5–500 ng/ml, the correlation coefficient was higher than 0.9990, the detection limit was 20.4–44.8 μg/kg, and the quantitation limit was 69.7–115.5 μg/kg. The recovery rate of TCs added to the chicken muscle samples was 84.7–106.3%, and that of RSDs was 0.4–4.5% (Weng et al., 2019). Rajaji and Chen prepared a chloramphenicol (CAP) amperometric sensor using Eu_2_O_3_ NPS-@rGO. The sensor can be used in honey and fresh milk samples, with a high recovery rate. It possesses high sensitivity and repeatability and can detect concentrations as low as 1.32 nM (Rajaji et al., 2019b). Main and Chen prepared a chloramphenicol (CAP) amperometric sensor using MoS_2_/f MWCNT nanocomposite. The sensor can be used in milk, honey, and powdered milk, with a high recovery rate. Under optimized working conditions, the nanocomposite film–modified electrode responds linearly to CAP in the concentration range of 0.08–1392 μM. The detection limit was obtained as 0.015 μM (±0.003). The electrode has a high level of selectivity in the large excess concentrations of interfering species. In addition, the modified electrode offers satisfactory repeatability, reproducibility, and stability (Govindasamy et al., 2017b). Vinoth et al. developed an almond-like structured SrMoO_4_ embedded on sulfur-doped–graphitic carbon nitride composites (SrMoO_4_/SGCN) using green methods for the electrochemical detection of CAP. The sensor can be used in river water samples, urine, and human blood serum with a high recovery rate. SrMoO_4_/SGCN/GCE impedance shows a lower resistance charge transfer (Rct), which helps favor the superior electrochemical detection of CAP. The SrMoO_4_/SGCN/GCE exhibits an ultralow detection limit of 1.5 nM for an extensive concentration range of 0.005 1316.8 M and high sensitivity is 9.619 AM/cm^2^ using the amperometric method (Vinoth et al., 2021). Govindasamy et al.developed a novel core-shell Bi_2_S_3_@GCN electrode material–modified SPCE using green methods for a highly sensitive and selective electrocatalytic detection of antibiotics. Under the optimal conditions of electrochemical analysis, the CPL sensor exhibited responses directly proportional to concentrations (a toxic chemical) over a range of 0.02 374.4 μM, with a nanomolar detection limit of 1.2 nM (signal-to-noise ratio S/N = 3) (Govindasamy et al., 2021). Wei’s study provides an effective tool for the selective and rapid detection of SM_2_ in food. A three-dimensional molecularly imprinted polymer (MIP) array electrochemical sensor was used to detect sulfadiazine (SM_2_) residues in food. Under optimized conditions, a wide linear range of 0.2–1000 ng/ml and a detection limit of 0.169 ng/ml (S/N = 3) were obtained. The recovery rate of the sensor is 92.3–102.23%, and the relative standard deviation is 2.27–4.10% (Wei et al., 2019). He and Wang developed a simple and sensitive tool for detecting fluoroquinolone residues in animal-derived foods. An MG–DSPE–HPLC method was used to extract seven types of FQ animal–derived food. The extraction method exhibited a high adsorption capacity (6800 ng) and enrichment coefficient (6879 times) for seven fluoroquinolones. The absorbent can be reused at least 40 times, the detection limit was within the range of 0.05–0.3 ng/g, and the recovery rate of the test samples (milk, chicken muscle, and egg) was 82.4–108.5% (He et al., 2017).

### Detection of Other Food Additives

Rajaji et al. developed a rapid detection of the feed additive drug (salbutamol) using bismuth telluride (Bi_2_Te_3_) decorated graphitic carbon nitride (GCN) nanostructures as a modified electrode for electrochemical sensing. A nanomolar limit of detection (1.36 nM) was calculated in a 0.05-M phosphate buffer (PB) supporting electrolyte (pH 7.0) using differential pulse voltammetry. The linear dynamic ranges concerning salbutamol concentration were 0.01 892.5 μM, and the sensor’s sensitivity was 36.277 μA μM^−1^cm^2^(Rajaji et al., 2021).

Umamaheswari et al. synthesized zinc sulfide nanospheres (ZnS NPs) encapsulated on reduced graphene oxide (RGO) hybrid by an ultrasonic bath (50 k*Hz*/60 W). As-prepared ZnS NPs@RGO hybrid was applied toward the electrochemical determination of caffeic acid (CA) in various food samples. The sensor can be used in red wine and soft drink samples with a high recovery rate. The proposed electrochemical caffeic acid sensor produces a wide linear range of 0.015–671.7 µM with a nanomolar level detection limit (3.29 nM) (Vinoth et al., 2021).

## Disadvantages

Graphene is an excellent new 2D material with an extensive application range (Yu et al., 2017; Zhang et al., 2014; Dresselhaus and Araujo, 2010). However, some disadvantages do exist. For example, the electrode material is limited by some application aspects when the graphene is separated from the dispersion liquid. Due to the influence of van der Waals forces, it tends to aggregate and accumulate during the drying process. This reduces the specific surface area. In addition, graphene hydrophobicity also limits the adsorption effect when detecting heavy metal ions in food. However, irreversible aggregation and repetition of graphene sheets limit their application on modified detection electrodes (Kim et al., 2010; Fan et al., 2010). Although multiple studies have shown that graphene has good biocompatibility, others have found that graphene possesses some biotoxicity. Graphene has a small particle size and quickly penetrates human skin, where it can interact with biological macromolecules such as proteins, lipids, and nucleic acids, generating certain biotoxicity (Singh et al., 2009; Makarucha et al., 2011). Consequently, graphene biosafety should be the focus of significant attention.

At present, the biodegradation of graphene materials mainly focuses on enzyme degradation. During body metabolic processes, the biological effect of hydrogen peroxide during substance oxidative decomposition is combined with active enzymes in the body to achieve the biodegradation of graphene materials. Differences in graphene (and its derivatives) structure, properties, and composition affect their degradation behavior. Using polyphase atom doping and surface functionalization, the physical and chemical properties of the materials are changed to affect the degradation process to regulate the enzymatic degradation of graphene materials. Some methods of obtaining graphene cannot meet research specifications due to dangerous pollution levels and low purity. Therefore, the development of green and environmentally friendly preparation methods is needed.

## Prospects

Based on existing electrochemical detection models, adding modified functional graphene-like materials to develop new, more reliable, rapid, and accurate detection methods and improving detection stability and limits has always been an essential topic in food detection. Although the commercialization of graphene materials is still in its infancy, the related long-term safety and environmental issues still need to be addressed. The physical and chemical properties of graphene have a decisive influence on biological outcomes, such as the effects of size, shape, surface charge, chemical composition, and surface modification on the toxicity of biofilm, which are still unknown. Furthermore, the active mechanisms are still unclear. Research on the mechanisms and biology of graphene interaction is still in its infancy at home and abroad. The physiological and biochemical processes caused by graphene’s impact on organisms have not been thoroughly studied. Therefore, it is urgent to study further the toxic effects and functional mechanisms of graphene biofilms.
